# The Unique Phylogenetic Position of a Novel Tick-Borne Phlebovirus Ensures an Ixodid Origin of the Genus *Phlebovirus*

**DOI:** 10.1128/mSphere.00239-18

**Published:** 2018-06-13

**Authors:** Keita Matsuno, Masahiro Kajihara, Ryo Nakao, Naganori Nao, Akina Mori-Kajihara, Mieko Muramatsu, Yongjin Qiu, Shiho Torii, Manabu Igarashi, Nodoka Kasajima, Keita Mizuma, Kentaro Yoshii, Hirofumi Sawa, Chihiro Sugimoto, Ayato Takada, Hideki Ebihara

**Affiliations:** a Laboratory of Microbiology, Faculty of Veterinary Medicine, Hokkaido University, Sapporo, Hokkaido, Japan; b Division of International Services, Global Institution for Collaborative Research and Education (GI-CoRE), Hokkaido University, Sapporo, Japan; c Division of Global Epidemiology, Hokkaido University Research Center for Zoonosis Control, Sapporo, Japan; d Laboratory of Parasitology, Faculty of Veterinary Medicine, Hokkaido University, Sapporo, Hokkaido, Japan; e Department of Virology III, National Institute of Infectious Diseases, Tokyo, Japan; f Hokudai Center for Zoonosis Control in Zambia, Hokkaido University Research Center for Zoonosis Control, University of Zambia, Lusaka, Zambia; g Division of Molecular Pathobiology, Hokkaido University Research Center for Zoonosis Control, Sapporo, Japan; h Laboratory of Public Health, Faculty of Veterinary Medicine, Hokkaido University, Sapporo, Hokkaido, Japan; i Division of Collaboration and Education, Hokkaido University Research Center for Zoonosis Control, Sapporo, Japan; j Department of Molecular Medicine, Mayo Clinic, Rochester, Minnesota, USA; University of Pittsburgh

**Keywords:** ancestral state, evolution, phylogenetic analysis, tick-borne phlebovirus

## Abstract

The recent emergence of novel tick-borne RNA viruses has complicated the epidemiological landscape of tick-borne infectious diseases, posing a significant challenge to public health systems that seek to counteract tick-borne diseases. The identification of two novel tick-borne phleboviruses (TBPVs), severe fever with thrombocytopenia syndrome virus (SFTSV) and Heartland virus (HRTV), as causative agents of severe illness in humans has accelerated the investigation and discoveries of novel TBPVs. In the present study, we isolated a novel TBPV designated Mukawa virus (MKWV) from host-questing Ixodes persulcatus females captured in Japan. Genetic characterization revealed that MKWV is a member of the genus *Phlebovirus* in the family *Phenuiviridae*. Interestingly, MKWV is genetically distinct from other known TBPVs and shares a most recent common ancestor with mosquito/sandfly-borne (insect-borne) phleboviruses. Despite its genetic similarity to insect-borne phleboviruses, the molecular footprints of its viral proteins and its biological characteristics define MKWV as a tick-borne virus that can be transmitted to mammals. A phylogenetic ancestral-state reconstruction for arthropod vectors of phleboviruses including MKWV based on viral L segment sequences indicated that ticks likely harbored ancestral phleboviruses that evolved into both the tick-borne and MKWV/insect-borne phlebovirus lineages. Overall, our findings suggest that most of the phlebovirus evolution has occurred in hard ticks to generate divergent viruses, which may provide a seminal foundation for understanding the mechanisms underlying the evolution and emergence of pathogenic phleboviruses, such as Rift Valley fever virus and SFTSV/HRTV.

**IMPORTANCE** The emergence of novel tick-borne RNA viruses causing severe illness in humans has complicated the epidemiological landscape of tick-borne diseases, requiring further investigation to safeguard public health. In the present study, we discovered a novel tick-borne phlebovirus from Ixodes persulcatus ticks in Japan. While its viral RNA genome sequences were similar to those of mosquito/sandfly-borne viruses, molecular and biological footprints confirmed that this is a tick-borne virus. The unique evolutionary position of the virus allowed us to estimate the ancestral phlebovirus vector, which was likely a hard tick. Our findings may provide a better understanding of the evolution and emergence of phleboviruses associated with emerging infectious diseases, such as severe fever with thrombocytopenia syndrome (SFTS) and Heartland virus disease.

## Abstract (precis)

The emergence of novel tick-borne RNA viruses causing severe illness in humans has complicated the epidemiological landscape of tick-borne diseases, requiring further investigation to safeguard public health. In the present study, we discovered a novel tick-borne phlebovirus from Ixodes persulcatus ticks in Japan. While its viral RNA genome sequences were similar to those of mosquito/sandfly-borne viruses, molecular and biological footprints confirmed that this is a tick-borne virus. The unique evolutionary position of the virus allowed us to estimate the ancestral phlebovirus vector, which was likely a hard tick. Our findings may provide a better understanding of the evolution and emergence of phleboviruses associated with emerging infectious diseases, such as severe fever with thrombocytopenia syndrome (SFTS) and Heartland virus disease.

## INTRODUCTION

Emerging and reemerging infectious diseases caused by arthropod-borne viruses (arboviruses) have become significant global public health concerns in recent decades. In particular, the emergence of mosquito-borne viruses such as Chikungunya virus (1), West Nile virus (2), and Zika virus (3), which have spread to new geographic locations with increased pathogenic potential, demonstrated that arboviruses pose ongoing and future global public health threats. This is due to the intricacies of host-vector-virus interactions and the capacity for viruses to adapt to new vectors or amplifying hosts, driving the selection of novel strains/variants with pandemic/epidemic potential (4–7).

In 2009, two novel tick-borne phleboviruses (TBPVs), severe fever with thrombocytopenia syndrome virus (SFTSV) (8) and Heartland virus (HRTV) (9), were recognized to cause severe and often fatal human illnesses resembling viral hemorrhagic fever with thrombocytopenia, lymphocytopenia, hemophagocytic syndrome, and multiorgan failure, resulting in fatality rates of up to 30%. HRTV was identified in the United States, whereas SFTSV was identified in East Asian countries, including China and Japan (10) and South Korea (11). Due to the similarities in the clinical features of TBPV diseases and severe cases of tick-borne bacterial/rickettsial diseases, such as human monocytic ehrlichiosis and human granulocyte anaplasmosis, newly emerging TBPVs have further complicated the epidemiological landscape of tick-borne infectious diseases, posing a significant challenge to public health authorities and health care providers in their efforts to prevent, diagnose, and treat tick-borne infectious diseases.

A phlebovirus that has enveloped spherical virus particles, containing tripartite and single-stranded RNAs (L, M, and S segments) with negative-sense and/or ambisense polarity, belongs to the genus *Phlebovirus* in the family *Phenuiviridae*, order *Bunyavirales* (formally known as the family *Bunyaviridae*) (12). Currently, the *Phenuiviridae* family consists of two species of TBPVs (i.e., *SFTS phlebovirus* and *Uukuniemi phlebovirus*) and several species of sandfly-borne and mosquito-borne viruses. Within the order *Bunyavirales*, the genus *Phlebovirus* is unique in that it contains human-pathogenic viruses transmitted by a wide range of arthropod vectors, either phlebotomine sandflies, mosquitos, or ticks. Recent advances in genome sequencing technology have allowed us to identify several tentative TBPV species/groups that are genetically distinct from the representative TBPV species *Uukuniemi phlebovirus*, revealing a greater diversity among TBPVs than among mosquito/sandfly-borne phleboviruses, which form a single clade with relatively low genetic divergence (13–17). Indeed, phylogenetic analyses have revealed that there are at least four TBPV lineages based on genetic/serological similarities (14): SFTSV/HRTV, belonging to the SFTS group and associated with hemorrhagic fever-like illness; Bhanja virus (BHAV) and Bhanja group viruses, associated with sporadic febrile illness cases; and Kaisodi virus (KSOV) and Uukuniemi virus (UUKV) in the Kaisodi and Uukuniemi groups, respectively, which have not been recognized as human pathogens. The mechanism(s) underlying the emergence of various TBPVs, especially two genetically related, highly pathogenic TBPVs (SFTSV and HRTV) on two different continents, however, has not yet been revealed.

In the present study, we successfully isolated a novel TBPV named Mukawa virus (MKWV) from Ixodes persulcatus in Japan. We have characterized the molecular and biological properties of the novel virus using virological and bioinformatics approaches and revealed that MKWV possesses the molecular and biological signatures of both tick-borne and mosquito/sandfly-borne phleboviruses. A better understanding of the unique evolutionary traits of MKWV will contribute to the identification of the molecular determinants responsible for the evolution of viruses in the genus *Phlebovirus* and the emergence of novel pathogenic phleboviruses, especially TBPVs.

## RESULTS

### Tick collection and isolation of MKWV.

**FIG 1:**
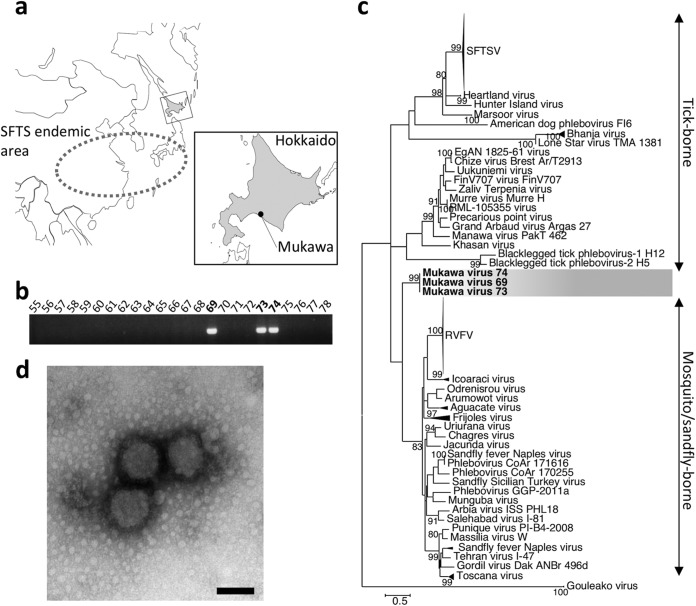
Detection and isolation of MKWV. (a and b) Ticks (Ixodes persulcatus) collected in Mukawa, Hokkaido, Japan (a), were genetically screened for tick-borne phleboviruses (TBPVs) by using the pan-TBPV RT-PCR (b). Numbers at top are sample number designations. (c) The nucleotide sequences of amplified DNA fragments were phylogenetically analyzed with those of other phleboviruses, and all MKWV fragments clustered together in the independent branch. (d) Virions of the MKWV isolate MKW73, harvested from the ISE6 tick embryonic cell supernatant, were morphologically analyzed using scanning electron microscopy. Bar, 100 nm. SFTSV, severe fever with thrombocytopenia syndrome virus; RVFV, Rift Valley fever virus.

The southwestern part of Japan is an area of endemicity of SFTS (10) (Fig. 1a). The RNA genome of SFTSV has been detected in multiple species of ticks in the area of endemicity, and the seroprevalence of SFTSV in wild animals, such as Shika deer and raccoons, has also been confirmed (18). To gain further insights into the ecology and epidemiology of TBPVs and to identify novel TBPVs other than SFTSV in Japan, we therefore collected ticks outside the SFTS area of endemicity (i.e., Hokkaido prefecture). In 2013, adult host-questing ticks were collected by the flagging method in Mukawa, Hokkaido, Japan (Fig. 1a), and tick species were identified morphologically. Total RNA was extracted from a total of 66 individual ticks and subjected to reverse transcription-PCR (RT-PCR) to amplify the phlebovirus L gene (14). Fragments of approximately 500 bp were amplified from three RNA samples from female I. persulcatus specimens (samples 69, 73, and 74) (Fig. 1b). Following nucleotide sequencing, a phylogenetic tree was constructed (Fig. 1c). Interestingly, the phylogenetic analysis of the partial L segment RT-PCR amplicons indicated that the all three RT-PCR products were derived from viruses belonging to the genus *Phlebovirus* that formed a cluster independent of any known phleboviruses. The detected phleboviruses have been tentatively designated MKWVs.

To isolate MKWVs, homogenates of RT-PCR-positive ticks were used to inoculate cultured cells (Vero E6, DH82, Huh7, and ISE6 cells) and newborn BALB/c mice. The replication of two isolates, MKW73 and MKW74, was confirmed by RT-PCR only in the supernatants of tick-derived ISE6 cells without cytopathic effects. Replication of MKWVs was not detected by RT-PCR in any mammal-derived cell lines or in newborn mice. The production of virions in the supernatant of ISE6 cells infected with the isolate MKW73 was confirmed by transmission electron microscopy (Fig. 1d). The virions of MKW73 are enveloped spherical particles (average diameter, 100 nm) with surface spike proteins, indicating morphological similarity with bunyaviruses (19).

### Gene structure/organization of MKWV.

For the removal of endogenous viruses from ISE6 cells (20, 21), MKW73 was passaged in Huh7 cells once, and the removal of endogenous viruses was confirmed by RT-PCR (data not shown). MKW73 RNA extracted from the supernatant of Huh7 cells was subjected to deep sequencing. The sequencing reads were *de novo* assembled into contigs, three of which were associated with phleboviruses according to a BLAST search of GenBank. After the sequencing of genomic RNA termini by rapid amplification of cDNA ends (RACE) and resequencing of regions with low-quality scores by Sanger sequencing, the complete nucleotide sequence of each segment of MKW73 was obtained; large (L), medium (M), and small (S) RNA segments were composed of 6,443, 3,327, and 1,907 nucleotides, respectively (Table 1).

**TABLE 1:** Genome structure of Mukawa virus

RNA segment	Length (base)	Protein(s)	ORF position (length) (bp)^a^	Terminal sequence (5′–3′)	
				5′	3′
L	6,443	RdRp	19–6312 (6,294)	ACACAAAGTCCG	GCGCCTTTGTGT
M	3,327	Gn and Gc	22–3243 (3,222)	ACACAAAGACCG	CCGTCTTTGTGT
S	1,907	N	36–776 (741)	ACACAAAGACCC	GGGTCTTTGTGT
		NSs	1871–852 (1,020)		

a In cRNA, not including stop codon.

The deduced amino acid sequences of the L and M segment open reading frames (ORFs) showed homology with those of L proteins (RNA-dependent RNA polymerase) and surface glycoproteins (polyproteins Gn and Gc) from known phleboviruses, respectively. The S segment of the isolate possessed two ORFs in ambisense. A BLAST search indicated that the deduced amino acid sequences of the ORFs were related to those of nucleocapsid proteins (N) and nonstructural proteins (NSs) from phleboviruses. The sequences of the L, M, and S genomic termini of MKW73 were identical to corresponding conserved sequences among phleboviruses (i.e., 3′-UGUGU and ACACA-5′), which may allow each segment of RNA to form panhandle structures. Thus, the genetic characteristics of MKWV clearly indicated that it is classified into the genus *Phlebovirus*.

### Phylogenetic characterization of MKWV.

**FIG 2:**
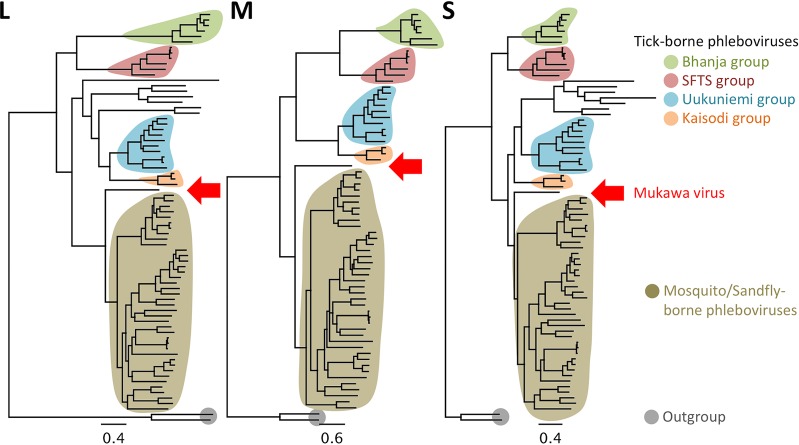
Phylogenetic analysis of MKWV RNA segments. Full-length sequences of RNA segments of MKWV were used to construct phylogenetic trees of the genus *Phlebovirus* using MrBayes. The background colors of branches indicate branches that are grouped together into biological groups. The positions of MKWVs in the tree are indicated by red arrows. Original trees with node names and posterior probability are provided as Fig. S1 to S3.

**FIG 3:**
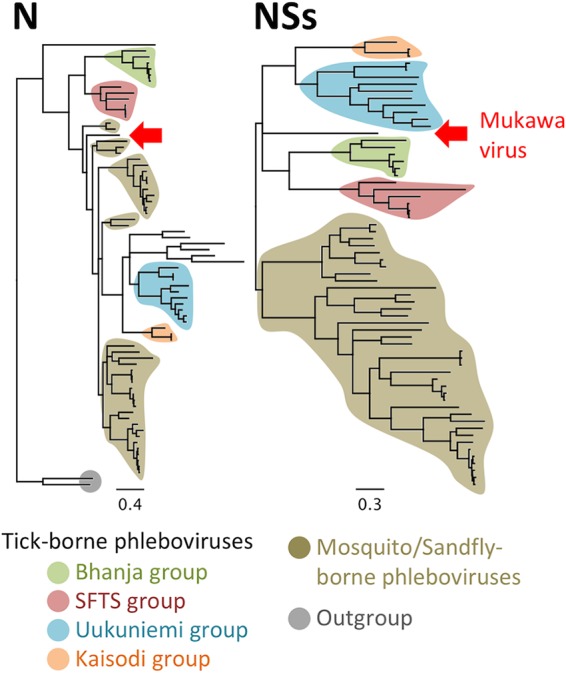
Phylogenetic analysis of MKWV N and NSs proteins. Deduced amino acid sequences of the MKWV nucleoprotein (N) and nonstructural protein (NSs) were used to construct phylogenetic trees of the genus *Phlebovirus* by using MrBayes. The background colors of branches indicate branches that are grouped together into biological groups. The positions of MKWV in the tree are indicated by red arrows. The original trees with node names are provided as Fig. S4 and S5.

To further determine the molecular relationship between MKWV and the other phleboviruses, including both tick-borne and mosquito/sandfly-borne phleboviruses, phylogenetic trees were constructed with MrBayes based on the nucleotide sequences of the L, M, and S segments (Fig. 2; see also Fig. S1, S2, and S3 in the supplemental material) and using Gouléako virus (22) and Cumuto virus (23) in the genus *Goukovirus* as an outgroup. The phylogenetic trees constructed from the full-length nucleotide sequences of the L, M, and S genome segments indicated that MKWV shares a most recent common ancestor with the mosquito/sandfly-borne phleboviruses, rather than with one of the four previously known groups of TBPVs or an additional tentative tick-borne phlebovirus group (Fig. 2). The phylogenetic trees based on the deduced amino acid sequences of L protein (Fig. S4) and glycoproteins (data not shown) have also been drawn with similar topologies that group MKWV together with mosquito/sandfly-borne phleboviruses. In the tree constructed from N proteins, the MKWV branch occurs between the Arumowot virus and the Salanga virus clusters, both of which are mosquito/sandfly-borne phlebovirus groups (Fig. 3 and S5), while the NSs proteins of MKWV were more closely related to those of TBPVs, rather than of mosquito/sandfly viruses (Fig. 3 and S6). Based on the different phylogenetic histories suggested by the different phylogenetic topologies between trees of N and NSs proteins of MKWV, the S segment nucleotide sequence was further investigated for evidence of recombination using multiple recombination detection tools; however, no reliable genetic recombination(s) in the S segment of MKWV has so far been found (data not shown).

10.1128/mSphere.00239-18.1 FIG S1  Phylogenetic analysis of MKWV L segment. The full-length sequence of the L segment of MKWV was used to construct a phylogenetic tree of the genus *Phlebovirus* using MrBayes. Posterior probability is indicated on each branch. Download FIG S1, PDF file, 0.1 MB. Copyright © 2018 Matsuno et al. 2018 Matsuno et al. This content is distributed under the terms of the Creative Commons Attribution 4.0 International license.

10.1128/mSphere.00239-18.2 FIG S2  Phylogenetic analysis of MKWV M segment. The full-length sequence of the M segment of MKWV was used to construct a phylogenetic tree of the genus *Phlebovirus* using MrBayes. Posterior probability is indicated on each branch. Download FIG S2, PDF file, 0.1 MB. Copyright © 2018 Matsuno et al. 2018 Matsuno et al. This content is distributed under the terms of the Creative Commons Attribution 4.0 International license.

10.1128/mSphere.00239-18.3 FIG S3  Phylogenetic analysis of MKWV S segment. The full-length sequence of the S segment of MKWV was used to construct a phylogenetic tree of the genus *Phlebovirus* using MrBayes. Posterior probability is indicated on each branch. Download FIG S3, PDF file, 0.1 MB. Copyright © 2018 Matsuno et al. 2018 Matsuno et al. This content is distributed under the terms of the Creative Commons Attribution 4.0 International license.

10.1128/mSphere.00239-18.4 FIG S4  Phylogenetic analysis of functional domains of MKWV L protein. The deduced amino acid sequence of the L protein of MKWV was used to align with the other L protein sequences. The multiple sequence alignment was manually edited to extract conserved regions, including functional domains that are conserved among bunyavirus RNA polymerases. The extracted alignment of 150-amino-acid sequences was used to construct a phylogenetic tree of the genus *Phlebovirus* using MrBayes. Posterior probability is indicated on each branch. Download FIG S4, PDF file, 0.1 MB. Copyright © 2018 Matsuno et al. 2018 Matsuno et al. This content is distributed under the terms of the Creative Commons Attribution 4.0 International license.

10.1128/mSphere.00239-18.5 FIG S5  Phylogenetic analysis of MKWV N protein. The deduced amino acid sequence of the N protein of MKWV was used to construct a phylogenetic tree of the genus *Phlebovirus* using MrBayes. Posterior probability is indicated on each branch. Download FIG S5, PDF file, 0.1 MB. Copyright © 2018 Matsuno et al. 2018 Matsuno et al. This content is distributed under the terms of the Creative Commons Attribution 4.0 International license.

10.1128/mSphere.00239-18.6 FIG S6  Phylogenetic analysis of MKWV NSs protein. The deduced amino acid sequence of the NSs protein of MKWV was used to construct a phylogenetic tree of the genus *Phlebovirus* using MrBayes. Posterior probability is indicated on each branch. Download FIG S6, PDF file, 0.1 MB. Copyright © 2018 Matsuno et al. 2018 Matsuno et al. This content is distributed under the terms of the Creative Commons Attribution 4.0 International license.

### Molecular characterizations of MKWV proteins.

**FIG 4:**
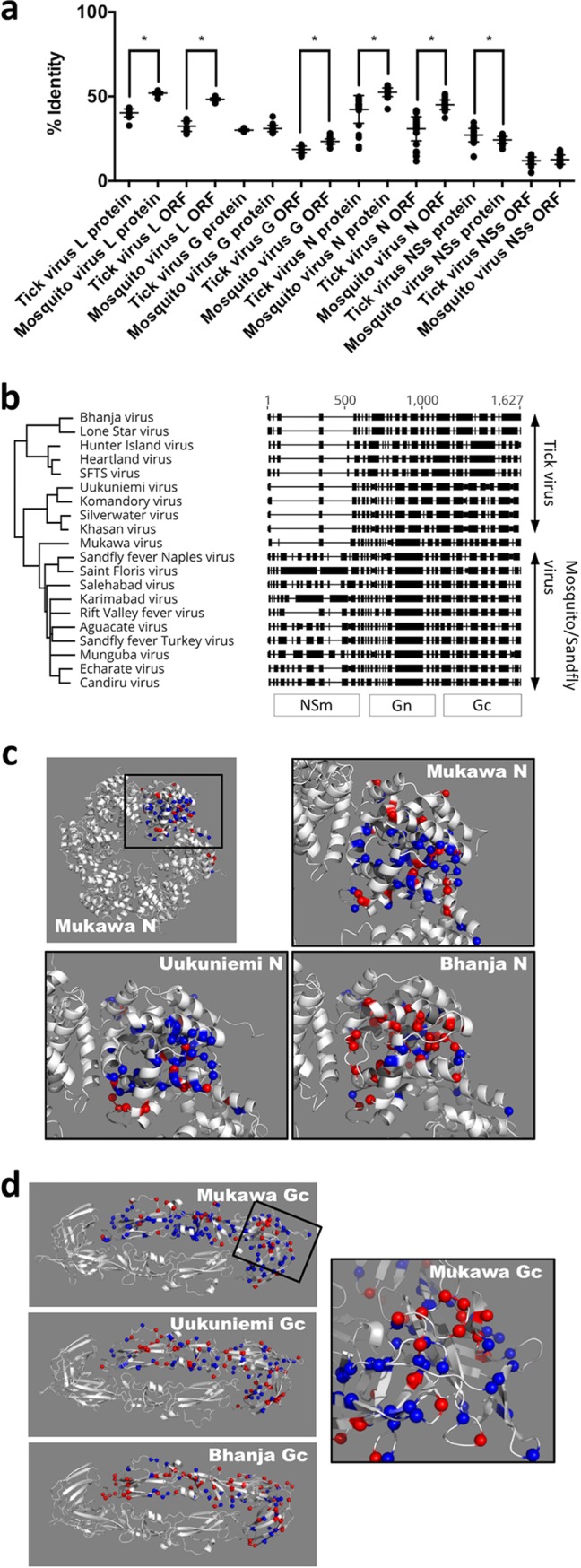
Molecular characterization of MKWV. (a) Nucleotide sequence identities of open reading frames or deduced amino acid sequence identities of viral proteins (RNA-dependent RNA polymerase [L], glycoprotein [G], nucleoprotein [N], or nonstructural protein [NSs]) were determined for the comparisons of MKWV with tick-borne phleboviruses (tick virus) and mosquito/sandfly-borne phleboviruses (mosquito virus). The mean identity of each group is presented as a horizontal bar. Significant differences between the identity values of tick virus comparisons and mosquito virus comparisons are indicated by asterisks (*P* < 0.05). (b) Alignments of deduced amino acid sequences of phlebovirus glycoproteins. Boxes indicate aligned amino acids, and bars indicate gaps in each sequence. (c and d) Structures of N protein (c) and membrane glycoprotein Gc (d) of three tick-borne phleboviruses, MKWV, Bhanja virus, and Uukuniemi virus, were predicted based on the crystal structures of RVFV proteins. Amino acid residues identical to those of RVFV and SFTSV are shown in blue and red, respectively. Amino acid residues shared with both RVFV and SFTSV are shown in dark gray. The inset view of the surface loop of domain II in the MKWV Gc is indicated by a black square.

To confirm the intermediate phylogenetic position of MKWV between TBPVs and mosquito/sandfly-borne phleboviruses, each nucleotide and protein sequence of MKWV was compared with those of other TBPVs or mosquito/sandfly-borne phlebovirus sequences (Fig. 4a). Except for the NSs sequences, which have the lowest identities among phlebovirus sequences, all the other MKWV sequences were more similar to the mosquito/sandfly-borne phlebovirus sequences than to those of the TBPVs. The sequence identities supported the phylogenetic analyses in which MKWV is more closely related to the mosquito/sandfly-borne phleboviruses than to the TBPVs.

The M segments of mosquito/sandfly-borne phleboviruses encode an additional nonstructural protein, NSm, upstream of Gn. While NSm is dispensable for proper Gn/Gc expression (24), the Rift Valley fever virus (RVFV) NSm protein can be associated with virulence (25, 26). A protein sequence alignment of glycoprotein precursors clearly showed that the MKWV M segment lacks the entire NSm region, similar to TBPVs (Fig. 4b). The high sequence similarity between the M segment ORF of MKWV and those of mosquito/sandfly-borne phleboviruses (Fig. 4a) therefore stems from similarities between the corresponding Gn and Gc regions.

For comparisons among the N and Gc proteins of phleboviruses, MKWV protein structures were predicted, as were the structures of the viral proteins of TBPVs, i.e., Bhanja virus and Uukuniemi virus. The protein models predicted in the present study mostly exhibited the same structural orientations, such as helix and sheets, as in the original N and Gc structures of RVFV proteins, respectively. In order to identify similar three-dimensional regions/domains that might not be observed in the sequence alignments between MKWV proteins and TBPV proteins, the amino acids in the predicted models were compared with those of a representative TBPV of the SFTS group, SFTSV, and a representative mosquito-borne phlebovirus, RVFV (Fig. 4c and d). As shown in the phylogenetic analysis, the N protein structures of MKWV and Uukuniemi virus were more closely related to that of RVFV than to that of SFTSV (Fig. 4c). In terms of the structure of the MKWV Gc protein, it was more similar to the structure of the RVFV Gc protein than to the other two Gc structures, and identical amino acids between MKWV and SFTSV seemed to predominate in domain II of Gc (Fig. 4d).

### Biological characterizations of MKWV.

**FIG 5:**
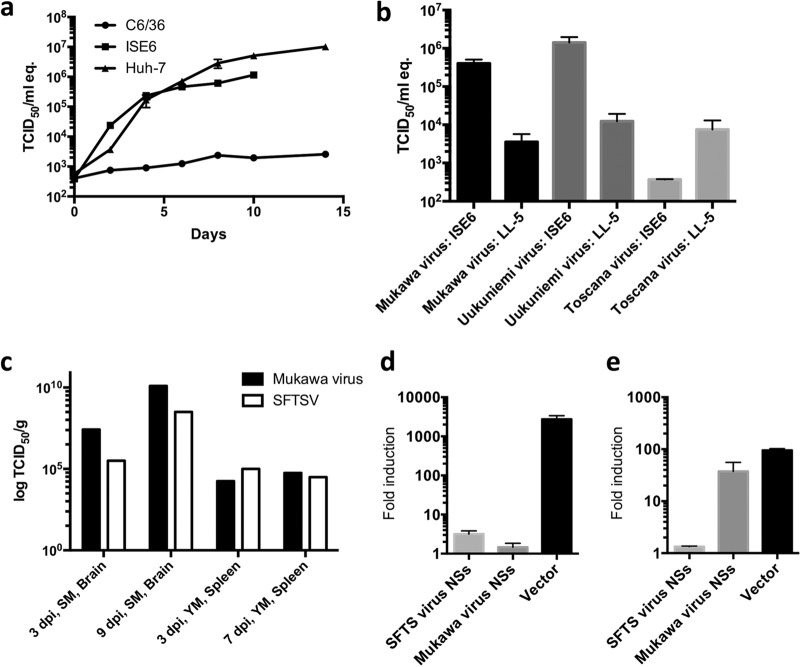
*In vitro* and *in vivo* replication of MKWV and interferon-antagonism activity of MKWV NSs. (a) MKWV isolate MKW73 was used to inoculate cells derived from human (Huh7), tick (ISE6), or mosquito (C6/36). The supernatant of each cell line was collected every 2 days from immediately after inoculation (day 0) to 14 days postinoculation (dpi). (b) Phleboviruses were inoculated into cells derived from tick (ISE6) or sandfly (LL-5), and the supernatant of each cell line was harvested at 7 dpi. RNA extracted from the supernatant was subjected to quantitative RT-PCR to calculate the viral titer. Each data point indicates the mean from triplicate experiments, and error bars indicate standard deviations (SDs). (c) MKWV MKW73 and SFTSV isolate YG1 were used to inoculate 1-day-old mice (SM) and 3-week-old mice (YM). Tissues were collected at 3, 7, and/or 9 dpi, and viral titers were calculated as the 50% tissue culture infectious dose (TCID_50_/gram). Error bars indicate SDs. (d and e) Luciferase activities under the control of the beta interferon promoter (d) and interferon-sensitive response element (ISRE) (e) were compared in NSs-expressing 293 cells stimulated by the expression of an activated form of RIG-I (d) and recombinant alpha interferon (e), respectively. All tests were done in triplicate, and the means ± SDs are shown.

To confirm the arthropod host(s) of MKWV, the replication of MKW73 was assessed in three different cell lines: human-derived Huh7 cells, tick-derived ISE6 cells, and mosquito-derived C6/36 cells (Fig. 5a). MKWV replication in each cell line was monitored for a maximum of 14 days until a monolayer of cells was disrupted. In the Huh7 and ISE6 cells, MKW73 replicated to maximum titers of 10^7^ 50% tissue culture infectious doses (TCID_50_)/ml equivalent and 10^6^ TCID_50_/ml equivalent, respectively. In contrast, C6/36 cells did not support the efficient reproduction of progeny infectious virions, and a slight increase of viral RNA was detected throughout the observation period. Next, the growth levels of MKWV, UUKV, and TOSV were compared in ISE6 cells and sandfly-derived LL-5 cells (Fig. 5b). ISE6 cells produced larger amounts of TBPVs (i.e., MKWV and UUKV) than LL-5 cells, which supported higher replication of sandfly-borne TOSV than ISE6 cells.

In addition, MKW73 was passaged in Huh7 cells and inoculated into newborn and 3-week-old C57BL/6J mice, and virus replication was monitored (Fig. 5c). All newborn mice infected with MKWV died by 9 days postinoculation (dpi). At 3 dpi, three infected mice from each group were sacrificed to examine viral replication in their brains. Productive viral replication was detected in the brains of MKWV-infected mice. In the young mice, productive replication of MKWV was also detected in the spleen at 3 and 7 dpi following intraperitoneal inoculation, indicating that MKWV replicated in mice and systematically spread within the body.

Phlebovirus NSs proteins have been recognized as significant virulence factors due to their ability to counteract host innate antiviral responses (27, 28). To determine the role of MKWV NSs proteins in their viral replication in human cells, inhibitory effects of NSs on the activities of the beta interferon promoter (Fig. 5d) or interferon-sensitive response element (ISRE) (Fig. 5e) were measured in human embryonic kidney 293 cells transiently expressing MKWV NSs and compared to those of cells expressing SFTSV NSs. MKWV NSs significantly blocked beta interferon promoter activity induced by RIG-I signaling but not ISRE activity, while SFTSV NSs inhibited both. During the transient expression of MKWV NSs in the cells, transcriptional shutoff, a significant function of RVFV NSs, was not detected.

### Ancestral-state reconstruction of the genus *Phlebovirus*.

**FIG 6:**
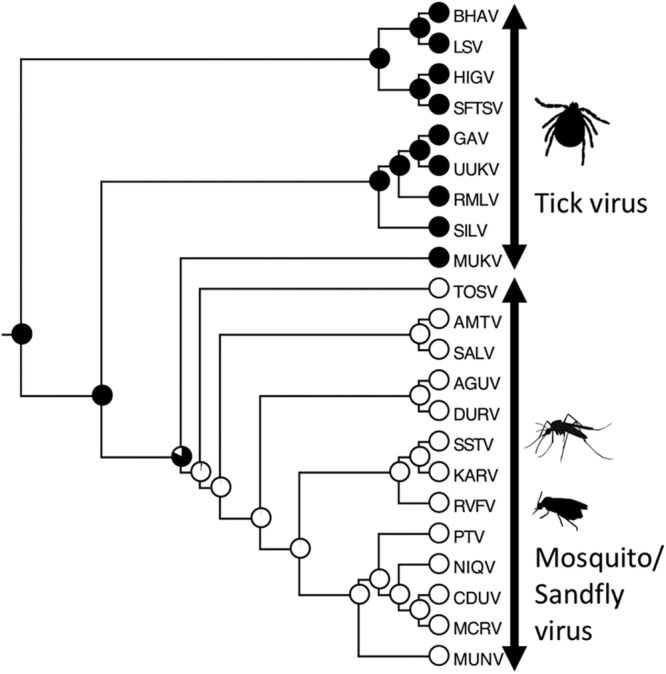
Phlebovirus ancestral-state reconstruction. The phylogenetic tree based on the nucleotide sequence of the L segment was used for ancestral-state reconstitution. Phleboviruses were divided into two states (insect-borne and tick-borne), and the state of each ancestor was predicted using the maximum likelihood method. The likelihood of each node or each ancestor is indicated by a pie chart (white, insect; black, tick). Abbreviations: BHAV, Bhanja virus; LSV, Lone Star virus; HIGV, Hunter Island virus; SFTSV, severe fever with thrombocytopenia syndrome virus; GAV, Grand Arbaud virus; UUKV, Uukuniemi virus; RMLV, RML-105355; SILV, Silverwater virus; MUKV (MKWV), Mukawa virus; TOSV, Toscana virus; AMTV, Arumowot virus; SALV, Salehabad virus; AGUV, Aguacate virus; DURV, Durania virus; SSTV, sandfly Sicilian Turkey virus; KARV, Karimabad virus; RVFV, Rift Valley fever virus; PTV, Punta Toro virus; NIQV, Nique virus; CDUV, Candiru virus; MCRV, Mucura virus; MUNV, Munguba virus.

For predicting the arthropod host(s) of ancestral phleboviruses, ancestral-state reconstruction was performed using the likelihood reconstruction method in Mesquite with a phylogenetic tree constructed based on nucleotide sequences of the L segment of the phleboviruses (Fig. 6). In the analysis, only the viruses with clearly identified arthropod vectors were used. The most recent ancestor of the MKWV and the mosquito/sandfly-borne phleboviruses, as well as more ancient ancestors of phleboviruses, was predicted to be harbored by ticks, not insects. The ancestor of the genus *Phlebovirus*, which was determined by using a defined outgroup (Fig. 2), was also predicted to be a tick-borne virus.

## DISCUSSION

**FIG 7:**
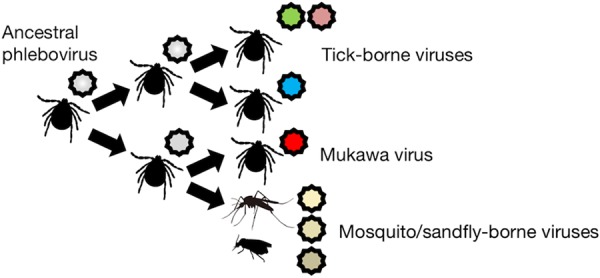
Hypothetical pathways of phlebovirus evolution. Based on our current knowledge of phleboviruses, the ancestor of phleboviruses is likely to be a tick-borne virus. The ancestral phlebovirus evolved into divergent viruses in ticks, and a variety of tick-borne viruses have been generated. MKWV and mosquito/sandfly-borne phleboviruses might be produced from the ancestor of Mukawa virus, and thus, less diversity can be observed among these insect-borne viruses than tick-borne phleboviruses.

The identification of SFTSV and HRTV stressed the potential public health threat of TBPVs. In addition to these novel pathogens, the identification of novel phleboviruses has been sporadically reported worldwide owing to the recent advancements and increasing availability of new sequencing technologies. Despite our increased knowledge of phleboviruses, their ecology and evolutionary dynamics remain poorly understood. In the present study, we identified a novel TBPV, designated MKWV, which was found to occupy a unique evolutionary position among phleboviruses. To date, the evolutionary history of TBPVs and mosquito/sandfly-borne phleboviruses has been completely unknown, though it was suspected that the ancestor of phleboviruses might have been an arthropod virus (15, 29). MKWV is the first TBPV to exhibit genetic similarity to mosquito/sandfly-borne phleboviruses. Despite the fact that MKWV shares common ancestry with and is phylogenetically closely related to mosquito/sandfly-borne phleboviruses, MKWV replicated at low levels in the mosquito-derived C6/36 cells and sandfly-derived LL-5 cells but replicated at higher levels in the tick (ISE6) and mammalian (Huh7) cells, like tick-borne UUKV, which was reported to replicate in Aedes aegypti mosquitos by experimental infections (30). Based on the discovery of this phylogenetically unique virus, the evolutionary history of phleboviruses with their vectors could be revisited with the stronger support values on trees. These results confirm that the ancestor of phleboviruses was a tick-borne virus that later generated various TBPVs and an ancestor of MKWV (Fig. 7). Further, the ancestor of MKWV evolved into mosquito/sandfly-borne phleboviruses and the current MKWV. Since more divergent insect-borne phleboviruses have not yet been reported in insect vectors, the “tick origin” hypothesis is consistent with our current knowledge of phleboviruses. Moreover, the Uukuniemi group viruses have been isolated from *Ixodes* hard ticks and *Argas* soft ticks (17), suggesting that the ancestral phlebovirus might retain the replication capability in hard ticks and soft ticks, as well as insects.

The topologies of phlebovirus trees were nearly identical among three RNA segments as well as three viral proteins (i.e., L, glycoprotein, and NSs), indicating the stable association among the three viral proteins. The topology of the N protein tree, however, in which some TBPVs, including MKWV, clustered with the mosquito/sandfly-borne phleboviruses, was unique. The difference between the N protein phylogeny and those of the other proteins and nucleotides may indicate a historical recombination event(s) or different selection pressures in the S segment, although no genetic recombination could be identified in the current analysis. Further discoveries of novel phleboviruses could shed additional light on the evolutional history of phleboviruses.

I. persulcatus, the tick species from which MKWV was isolated, is a common tick distributed across the Hokkaido prefecture and the northern part of the main island of Japan. I. persulcatus infests humans and animals, frequently resulting in cases of Lyme disease in Hokkaido (31). While several studies have been conducted on the bacterial pathogens carried by this tick species (32–34), there have previously been no reports of diseases indicating associations with viral pathogens. MKWV is likely to be transmitted to mammals through tick bites and to establish infections, as suggested by the growth of MKWV in a human-derived cell line and mice and by the anti-innate immune function of MKWV NSs in human cells. Based on these findings, it is conceivable that wild animals such as local deer and rodents may be infected with MKWV through tick bites and act as intermediate hosts, similar to the way in which SFTSV infects wild animals and livestock in regions of endemicity (35–37). Furthermore, the replication competence of MKWV in human-derived Huh7 cells at 37°C and ISE6 cells at 34°C indicates the potential of MKWV to replicate in both arthropod and mammalian hosts (29). A serological study of MKWV in humans and animals is thus warranted for furthering our understanding of the epidemiology of the virus. Moreover, it is highly plausible that a large number of phleboviruses remain unknown, and further intensive studies may reveal the evolutional mechanism(s) of the emergence of human-pathogenic phleboviruses.

## MATERIALS AND METHODS

### Tick collection and sample preparation.

Adult host-questing ticks were captured using the flagging method in Mukawa, Hokkaido, Japan (Fig. 1a) (latitude 42.61 north, longitude 141.95 east) in May 2013. Collected ticks were morphologically identified as I. persulcatus, Ixodes ovatus, Haemaphysalis japonica, or Haemaphysalis megaspinosa under a stereomicroscope. Ten males and 10 females of I. persulcatus, 12 males and 12 females of I. ovatus, three females of H. japonica, and 10 males and 10 females of H. megaspinosa were used in the following experiments. Each tick was homogenized twice with 100 µl of plain Dulbecco’s modiﬁed Eagle’s medium (DMEM) using a homogenizer (Tomy Seiko) at 3,000 rpm. Total RNAs were extracted from 50 µl of the homogenates by using the blackPREP tick DNA/RNA kit (Analytik Jena) according to the manufacturer’s protocol, and remaining lysate samples were stored at −80°C until use for virus isolation.

### One-step RT-PCR.

We utilized a previously reported one-step RT-PCR system that detects a wide-range of TBPVs from RNA samples from collected ticks (14). Briefly, one-step RT-PCR was performed with the PrimeScript One Step RT-PCR kit, version 2 (Dye Plus) (TaKaRa), with 1 µl of total tick RNA and 4 pmol of primers, HRT-GOUL2759F (5′-CAGCATGGIGGIYTIAGRGAAATYTATGT-3′) and HRT-GOUL3276R (5′-GAWGTRWARTGCAGGATICCYTGCATCAT-3′) in 10 µl of reaction solution with the following incubation program: 50°C for 30 min; 94°C for 2 min; 40 cycles of 94°C for 30 s, 55°C for 30 s, and 72°C for 30 s; and 72°C for 5 min. Amplified RT-PCR products were purified and sequenced with a 3130 Genetic Analyzer (ABI, Thermo Fisher Scientific).

### Cells and viruses.

Vero E6 (African green monkey kidney), Huh7 (human hepatocellular carcinoma), and DH82 (canine macrophage) cells were grown in DMEM supplemented with fetal calf serum (FCS). ISE6 (Ixodes scapularis embryo) cells were grown in modified L-15B medium supplemented with 10% FCS and 5% tryptose phosphate broth (Sigma) at 34°C (38). C6/36 (Aedes albopictus larva) cells were grown in minimal essential medium (MEM) supplemented with 10% FCS and l-glutamine at 28°C. SFTSV (strain YG1, kindly provided by Ken Maeda, Yamaguchi University, Japan) was propagated in Vero E6 cells (10).

### Virus isolation.

Tenfold-diluted tick lysates in DMEM supplemented with 10% FCS, 2 mM l-glutamine, 50 U/ml penicillin, 50 µg/ml streptomycin, and 25 µg/ml gentamicin (Sigma) were filtered and used to inoculate cultured cells. Cells were cultured for 14 days, and RNAs were purified from supernatants using the QIAamp viral RNA minikit (Qiagen). Isolates from two I. persulcatus samples were tentatively designated MKWVs. Blind passages were carried out once or twice. Virus shedding in the supernatants was confirmed by RT-PCR. Filtrates were also used to inoculate newborn BALB/c mice intracerebrally. The mice were observed for manifestations of clinical signs. At 14 dpi, mice showing no clinical sign were sacrificed, and their brains were sampled. Viral replication in brains was examined using RT-PCR.

### Electron microscopy.

The supernatant of TBPV-positive cells was harvested and pelleted by ultracentrifugation (27,000 rpm, 90 min) through 25% sucrose in phosphate-buffered saline (PBS). Virions were resuspended in PBS and adsorbed to collodion-carbon-coated copper grids. Virions negatively stained with 2% phosphotungstic acid solution (pH 5.8) were examined under an H-7650 transmission electron microscope (Hitachi) at 80 kV.

### Determination of full-length viral genome sequences.

Viral RNA extracted from purified virions was subjected to double-stranded cDNA synthesis using a cDNA synthesis kit (Moloney murine leukemia virus [M-MLV] version; TaKaRa). Pyrosequencing analysis was performed on a 454 Genome Sequencer Junior (GS-Junior; Roche) according to the manufacturer’s protocol. After trimming low-quality reads, the resulting reads were *de novo* assembled using Newbler (Roche) with default parameters. Both the 5′ and 3′ terminus regions of each segment were amplified by the RACE method (39), and the complete nucleotide sequence of each segment was obtained.

### Multiple sequence alignment and phylogenetic analysis.

The nucleotide sequences of the L gene fragments of approximately 500 bp amplified by RT-PCR were aligned with representative sequences from other known phleboviruses available from GenBank by using MUSCLE as implemented in MEGA, version 6 (40). Multiple sequence alignments were modified manually to correct unreliable alignments. Phylogenetic trees were constructed using the maximum likelihood (ML) method. For ML analysis, the Tamura-Nei model with gamma-distributed invariant sites (G+I) built into MEGA 6 was used. The robustness of each node was tested by 1,000 bootstrap replicates. The full-length nucleotide sequence of each RNA segment and deduced amino acid sequence of each viral protein of MKWV and the representative phleboviruses were subjected to the multiple sequence alignment using MEGA as well as Clustal Omega (41) as implemented in CLC Main Workbench (Qiagen). The aligned sequences were used for phylogenetic tree construction by using MEGA (ML and neighbor-joining methods) and MrBayes 3.2.2 (42) as a plug-in of Geneious (Biomatters Ltd.) with the GTR+G+I substitution model. The trees obtained by three independent methods were compared with each other to confirm the same topologies. Ancestral-state reconstruction was performed with Mesquite (43).

### Experimental infection of mice with TBPVs.

MKWV (5.0 × 10^4^ TCID_50_) and SFTSV (3.6 × 10^4^ TCID_50_) were intracerebrally inoculated into nine and six newborn mice (C57BL/6J), respectively. Three mice of each group were sacrificed at 3 dpi, and their brains were sampled. The remaining mice were observed for their clinical symptoms. MKWV (2.5 × 10^5^ TCID_50_) and SFTSV (1.8 × 10^5^ TCID_50_) were intraperitoneally inoculated into 11 3-week-old C57BL/6J mice. Three mice were sacrificed at 3 and 7 dpi, and their brains, kidneys, lungs, livers, and spleens were aseptically collected. The remaining mice were observed for clinical signs, and their body weights were recorded for 14 days. Viral replication in each tissue was examined as described above, and the titers were calculated as TCID_50_ per gram of tissue. Experimental infections were carried out in the biosafety level 3 facility at the Hokkaido University Research Center for Zoonosis Control, Japan, strictly according to the Guidelines for Proper Conduct of Animal Experiments of the Science Council of Japan. The protocol was approved by the Hokkaido University Animal Care and Use Committee. All efforts were made to minimize suffering.

### Growth kinetics of MKWV in various cells.

MKWV was inoculated into Huh7, ISE6, and C6/36 cells at a multiplicity of infection (MOI) of 1. After a 1-h incubation to allow viruses to attach, cells were rinsed once with their original medium and incubated for up to 14 days in the same medium with 10% FCS. Supernatants were collected every 2 days after the inoculation (0 to 14 days), and RNAs were extracted using TRIzol LS reagent (Thermo Fisher Scientific) according to the manufacturer’s protocol. Viral RNAs in the supernatant were quantified as equivalent to TCID_50_ by using real-time PCR with the KAPA Probe Fast qPCR kit (KAPA Biosystems) (primer and probe sequences available upon request).

### Innate immune signaling inhibition assays.

293 cells maintained in DMEM-10% FCS were transiently transfected with an expression plasmid of MKWV NSs-FLAG or SFTSV NSs-FLAG together with a reporter plasmid (pI125luc or pISRE-Luc) expressing firefly luciferase under the control of the beta interferon promoter or ISRE, respectively, and a control-luciferase-expressing plasmid, pGL4.75[hRluc/CMV] (Promega). The cells transfected with pI125luc were also transfected with an expression plasmid of the human RIG-I CARD domain (pEF-BOS RIG-IN) to activate the interferon-production signaling. The cells transfected with pISRE-Luc were treated with recombinant human alpha interferon (R&D Systems) 6 h prior to harvest. Cells were lysed at 24 h posttransfection, and the luciferase activity was measured by using the Dual-Glo luciferase assay system (Promega) with a Modulus microplate luminometer.

### Accession number(s).

The pyrosequencing data and complete genome sequences were deposited in the Sequence Read Archive (SRA) under accession number DRA002645 (raw reads) and in EMBL/GenBank/DDBJ under accession numbers LC063768 to LC063770 (complete genome sequences).
