# Supplementary figures and images for: The Unique Phylogenetic Position of a Novel Tick-Borne Phlebovirus Ensures an Ixodid Origin of the Genus Phlebovirus
Source: mSphere. 2018 Jun 13;3(3):e00239-18. doi: 10.1128/mSphere.00239-18 (PMC6001614; doi:10.1128/mSphere.00239-18)

Fig. S1 Matsuno et al.

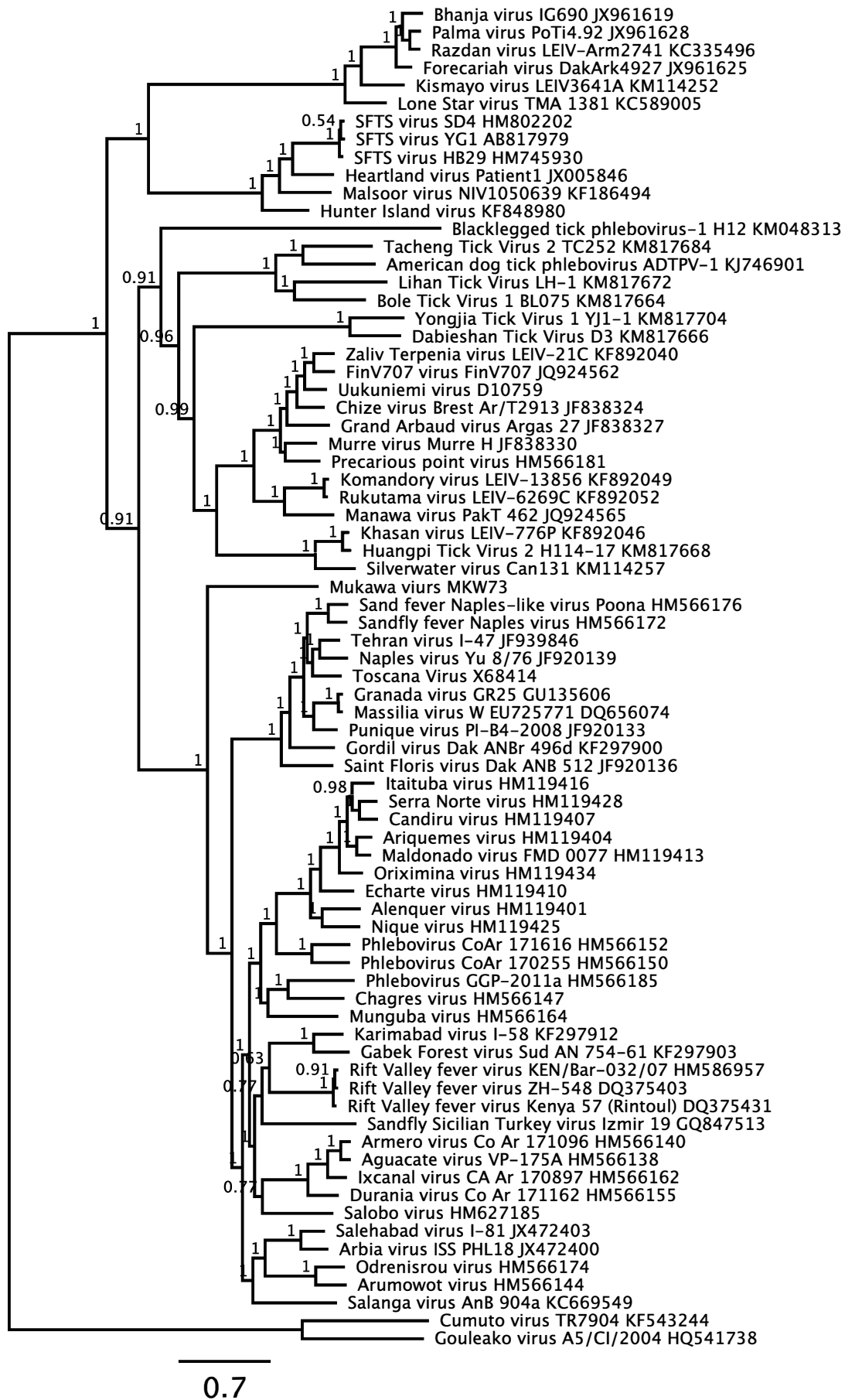

Supplement: FIG S1 [file sph003182570sf1.pdf]

Fig. S2 Matsuno et al.

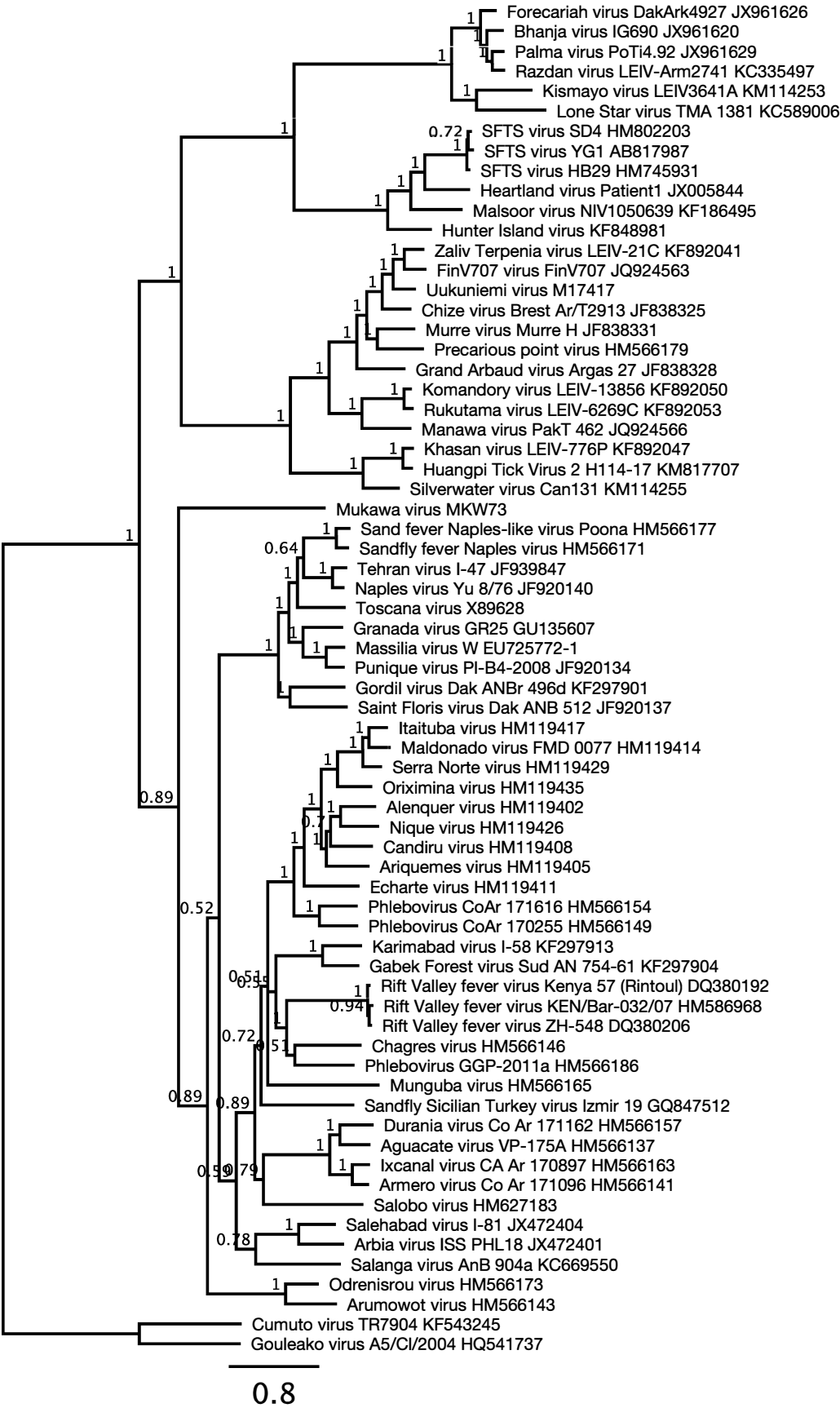

Supplement: FIG S2 [file sph003182570sf2.pdf]

Fig. S3 Matsuno et al.

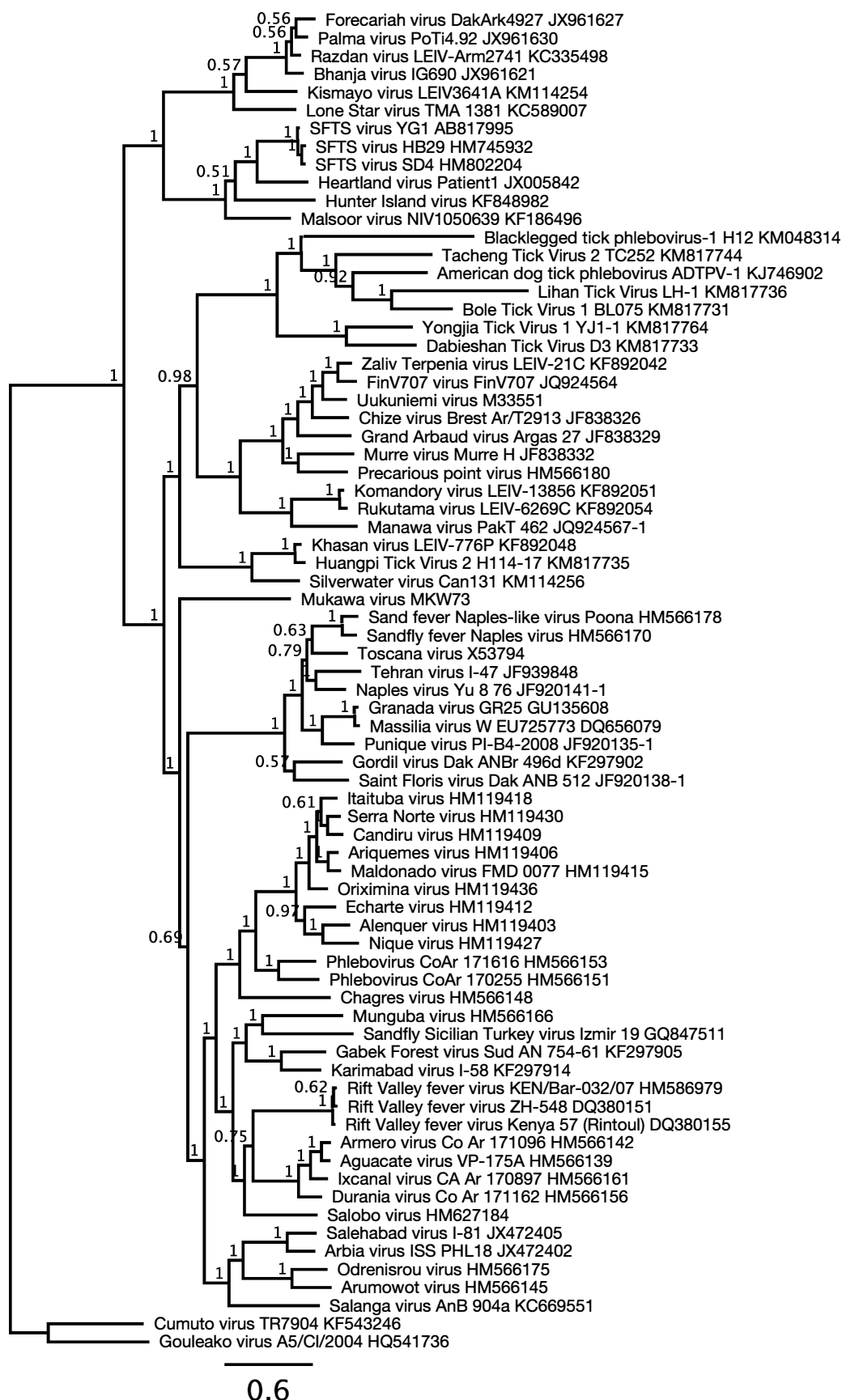

Supplement: FIG S3 [file sph003182570sf3.pdf]

Fig. S4 Matsuno et al.

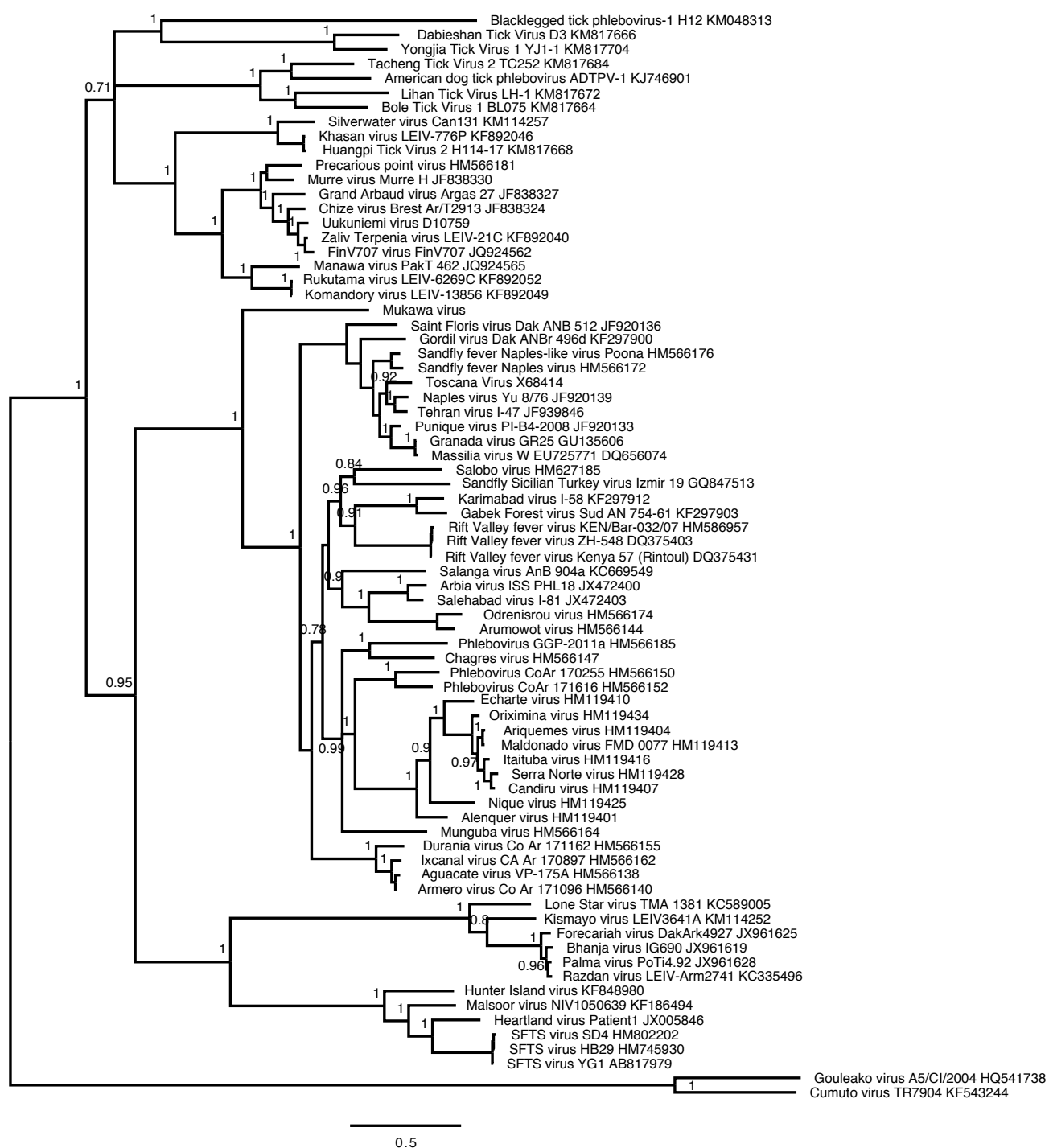

Supplement: FIG S4 [file sph003182570sf4.pdf]

Fig. S5 Matsuno et al.

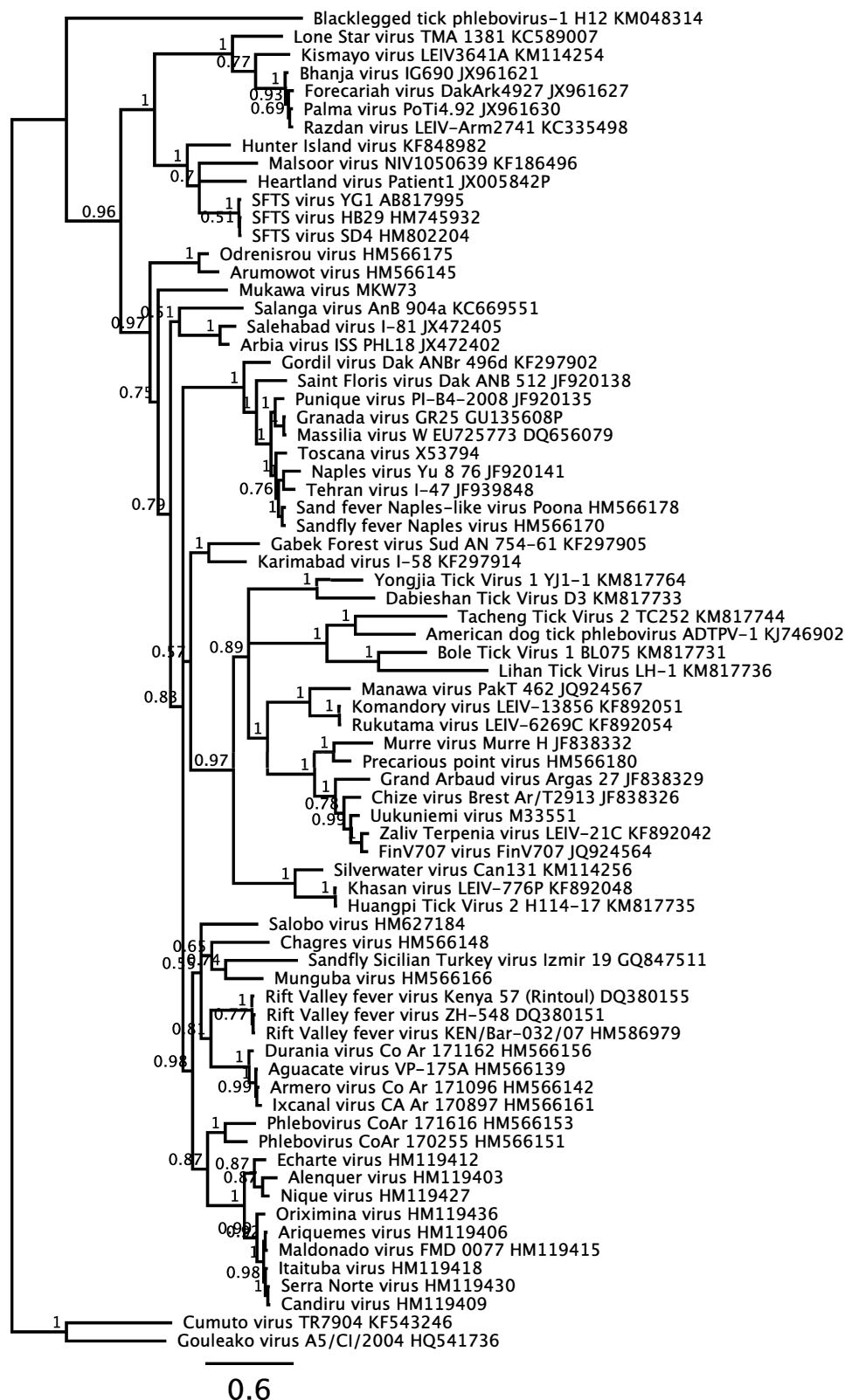

Supplement: FIG S5 [file sph003182570sf5.pdf]

Fig. S6 Matsuno et al.

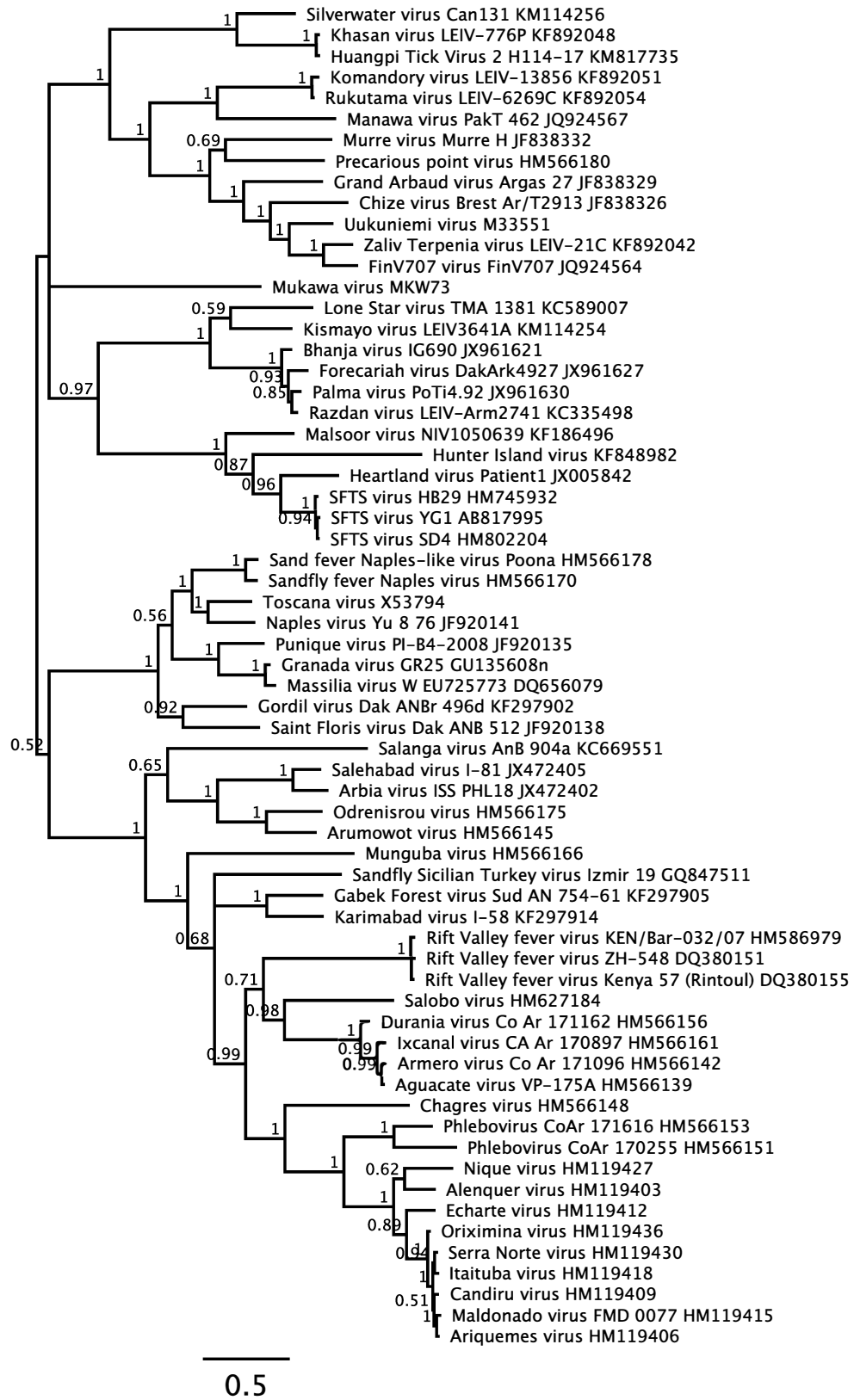

Supplement: FIG S6 [file sph003182570sf6.pdf]
